# Disease and Treatment-Related Sequelae in Patients with Complex Jugulotympanic Paraganglioma

**DOI:** 10.3390/jcm7030051

**Published:** 2018-03-10

**Authors:** Ali Harati, Rolf Schultheiß, Stefan Rohde, Thomas Deitmer

**Affiliations:** 1 Neurosurgical Department, Klinikum Dortmund, 44145 Dortmund, Germany; rolf.schultheiss@klinikumdo.de; 2 Department of Radiology and Neuroradiology, Klinikum Dortmund, 44145 Dortmund, Germany; stefan.rohde@klinikumdo.de; 3 Department of ENT, Klinikum Dortmund, 44145 Dortmund, Germany; thomas.deitmer@klinikumdo.de

**Keywords:** jugular foramen, microsurgery, jugulotympanic paraganglioma, facial nerve, lower cranial nerves, internal carotid artery, vertebral artery

## Abstract

Background: Jugulotympanic paraganglioma (JTP) are benign, high-vascularized lesions that frequently invade the jugular foramen, temporal bone, the upper neck, and the posterior fossa cavity, resulting in a wide variety of clinical symptoms. Methods: In this retrospective study, we assess the clinical symptoms and discuss the individual multidisciplinary treatment and outcome of 22 patients with JTP. Results: In 12 patients, a hearing deficit was the presenting symptom, whereas pulsatile tinnitus and otalgia were present in six and four patients respectively. Facial nerve involvement was seen in six patients (three HB Grade 1–2 and three HB Grade 4–6). Four patients presented with lower cranial nerve impairment. Rare symptoms were ataxia caused by brainstem compression and papilledema due to cerebral sinus obstruction. A new or worsening of the preoperative facial nerve or lower cranial nerve function occurred in two and four patients respectively. Conclusion: The treatment strategy and the surgical approach for JTP should be tailored to the tumor extension and the patient’s clinical symptoms.

## 1. Introduction

Paragangliomas (PGLs), also known as chemodectomas, have their origin in paraganglia of the chemoreceptor system. PGLs can be divided into parasympathetic PGLs (head and neck PGLs) and sympathetic PGLs. Especially malignant and young-onset PGL can be involved in PGL syndromes (PGL 1–5) caused by SDHx mutations. PGLs arise from the carotid and aortic bodies and the glomus jugulare but can also be located in the ganglion nodosum of the vagus nerve, the middle ear, and other sites throughout the body. Jugulotympanic paraganglioma (JTP), also known as glomus jugulare tumors, arise from a small group of cells in the adventitia of the jugular bulb. Despite their suspected benign origin, they represent locally aggressive, destructive neoplastic lesions, with frequent invasion of the middle ear, the temporal bone, the upper neck, and through the jugular foramen, into the posterior cranial fossa. Based on their location, size, and extent, JTP can be classified by Fisch into four categories (Table 1).

In patients with JTP, the clinical signs and sequelae relate to the location of the tumor and invasion of the neural and vascular structures. The aim of the present study is to analyze the clinical features and treatment-related sequelae of patients with complex JTP.

## 2. Patients and Methods

### 2.1. Patients

Sixty-eight patients with head and neck PGL were treated in our institution between January 2003 and November 2017. Of these patients, 22 (20 women, two men) presented with complex JTP and were referred to our department for surgery. Patients’ files and images were reviewed retrospectively. Patient data were collected throughout the follow-up period. Patient characteristics are displayed in Table 2.

### 2.2. Clinical Evaluation

**Figure 1:**
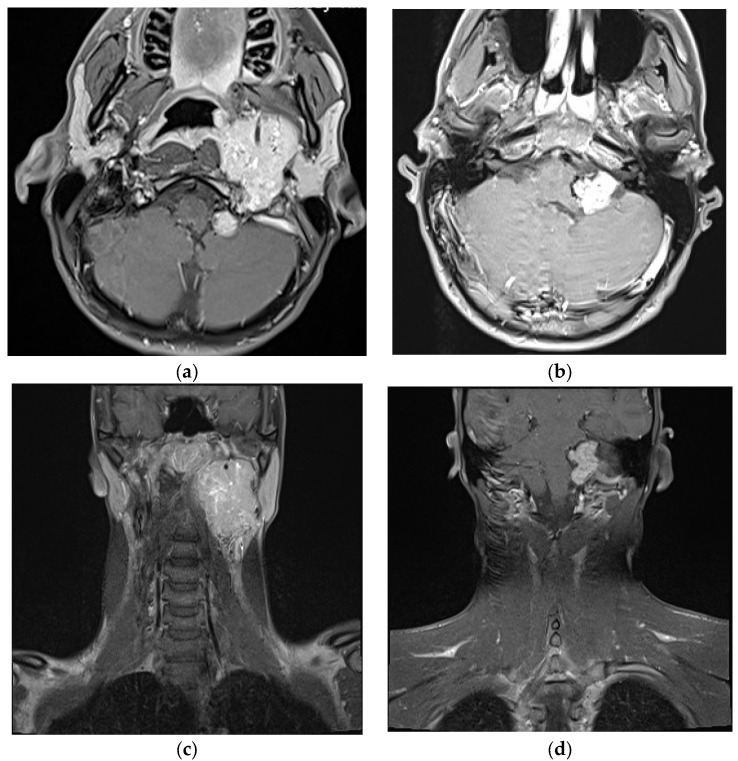
(**a**–**d**) T1 contrast enhanced transversal MRI demonstrating the tumor growing from the jugular foramen into the posterior cavity and the upper neck.

**Figure 2:**
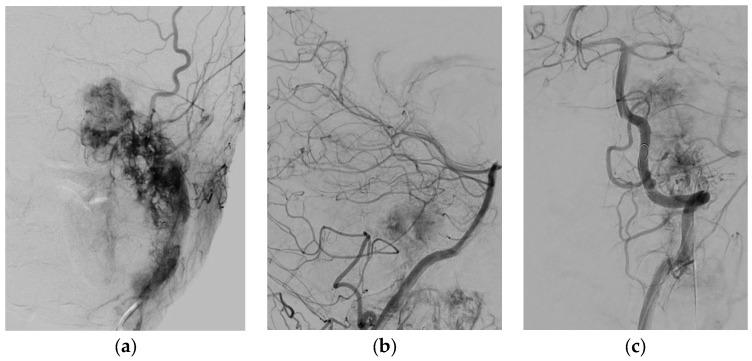
(**a**) Superselective DSA of the external carotid artery of the high vascularized tumor; (**b**,**c**) DSA demonstrate feeding vessels of intradural tumor from the vertebral artery.

All patients underwent complete clinical and neurological evaluation. Facial nerve function was assessed before and after treatment and at each follow-up using the House-Brackmann scale. Dysphagia and vocal cord function were evaluated to assess lower cranial nerve involvement. Motor examination, including the strength of all extremities, coordination, and gait, were examined routinely to assess brainstem and cerebellar affection. Additionally, level of consciousness, orientation, memory, and other signs of elevated intracranial pressure (ICP) were evaluated in selected cases. Ophthalmological examination was performed in case of visual impairment, diplopia, or nystagmus. Genetic testing was performed in all but four patients who were older than 70 years. One young patient (see Figure 1 and Figure 2) had SDH mutation (SDHB). Assessment for catecholamine secretion by the tumor was performed in all patients. In the same patient with SDH mutation a hypersecreting tumor was identified and additionally an abdominal computed tomographic scan was obtained to rule out an adrenal source of catecholamine secretion.

### 2.3. Radiologic Evaluation

High-resolution bone window computed tomography (CT) studies, essential for visualizing of bone destruction and tumor involvement of the mastoid air cells, the jugular foramen, the internal auditory canal, and the cochlea, were obtained from every patient. Additionally, magnetic resonance imaging (MRI) was performed in all patients to visualize the relationship of the tumor to the jugular foramen, the brainstem, the vertebral artery, and the cranial nerves. T-2 weighted MR-imaging studies were used to assess fourth ventricle distortion and hydrocephalus.

All patients received cranial digital subtraction angiography (DSA). DSA is essential to assess feeding vessels and venous drainage of the tumor. When imaging reveals carotid or vertebrobasilar artery involvement or intracranial extension, we routinely employed four-vessel angiography to define the vascularity of the tumor, extent of internal carotid and vertebral artery involvement, survey for multifocal disease, and partially devascularize the tumor through selective embolization of accessible feeding vessels. Together with MR angiography, DSA represents a valuable diagnostic tool in detecting transverse or sigmoid sinus hypoplasia, jugular bulb obstruction by the tumor, and extracranial venous drainage.

### 2.4. Treatment Modalities

Endovascular occlusion of JTP was performed by superselective catheterization of multiple, small feeding branches, and anastomosis between the internal carotid artery, the external carotid artery, and the vertebral artery. Transarterial embolization was performed by ONYX, detachable coils, or both [1,2,3].

In our institution, the tumor was mainly approached through a combined retro- and infralabyrinthian approach, without transposition of the facial nerve. Due to the highly variable extension of especially large TJP, the surgical approach was tailored individually based on the tumor extension. The microsurgical techniques were described in detail in a recent article [1].

Radiotherapy was recommended either because the risk of surgery-associated morbidity was high or because of residual tumor that remained following surgical resection. Beside stereotactic radiosurgery, intensity modulated radiotherapy was the preferred standard treatment modality [4]. Patients received 45 Gy in 25 once-daily fractions in a continuous course over 5 weeks.

## 3. Results

### 3.1. Clinical Data

In 12 patients, a hearing deficit was the presenting symptom. Sudden sensorineural hearing loss occurred in one patient, while two patients developed surdity within several months. Pulsatile tinnitus and otalgia were present in six and four patients respectively.

Mild facial nerve involvement was seen in three patients (HB Grade 1–2) and progressive facial nerve palsy was present in three patients (HB Grade 4–6).

Four patients presented with affection of cranial nerves IX and X. The main symptoms were hoarseness and dysphagia. Other symptoms related to lower cranial nerve deficits were rough or breathy voice quality, vocal fatigue, glottal insufficiency, globus sensation, and throat pain.

One patient presented with accessory nerve involvement and exhibited signs of diminished muscle mass, fasciculation, and partial paralysis of the sternocleidomastoid and trapezius muscles. Interruption of the nerve supply to the sternocleidomastoid muscle resulted in an asymmetric neckline, while weakness of the trapezius muscle produced a drooping shoulder and a weakness of the forward elevation of the shoulder.

Three patients developed tongue atrophy and deviation to one side caused by hypoglossal nerve palsy. These patients additionally complained about slurred speech.

Rare symptoms were ataxia caused by cerebellar and brainstem compression and blurring of vision together with visual obscurations caused by papilledema due to cerebral sinus obstruction (Illustrative case 2).

### 3.2. Treatment and Outcome

DSA of the intra- and extracranial vessels was performed in all patients. Two patients demonstrated hypoplasia of the transverse sinus contralateral to the tumor site. Resection of the tumor with the affected sigmoideus sinus bore the risk of elevated intracranial pressure due to venous stasis. Therefore, we did not offer surgery in these cases. In three other patients, surgical resection was not offered due to high age or poor clinical condition. Palliative embolization to treat pulsatile tinnitus and radiotherapy were performed without any clinical deterioration.

Surgical resection after endovascular embolization was performed in 17 patients. Radical tumor resection was achieved in twelve patients. Near-total resection was achieved in the remaining five patients. Additional postoperative radiation therapy was offered in these cases. There was no operative and postoperative mortality. There were no major complications, such as large vessel injury, intracranial bleeding, or ischemia. There were no cases of postoperative ataxia or temporary limb palsy.

In three patients with initial facial nerve palsy House-Brackmann (HB) grade IV due to tumor invasion of the fallopian canal, the preexisting facial nerve palsy remained unchanged. Two patients developed postoperatively temporary facial nerve palsy HB grade V, which improved to grade III during long-term follow-up. In these cases, a combined upper eyelid loading and lower lid canthopexy were performed to provide symptomatic relief from corneal exposure with a reasonable cosmetic appearance.

In nine patients with the preoperative hearing deficit, three patients had complete sensineural hearing loss postoperatively, whereas three patients had improvement of hearing function in long-term follow-up. Pulsatile tinnitus and otalgia resolved in nearly all patients.

A new deficit of the lower cranial nerves occurred in four patients. A prophylactic nasogastric feeding tube was needed in the immediate postoperative period for those patients. However, two patients developed severe aspiration pneumonia and required tracheotomy and temporary percutaneous endoscopic gastrostomy. None of the patients with preoperative lower cranial nerve deficits experienced any complications, such as aspiration or pneumonia. Among all patients, pre- and post-operative lower cranial nerve impairment remained compensated in four out of seven patients during long-term follow-up. Three patients with vocal cord palsy suffered chronic hoarseness and were treated with silicone thyroplasty. Long-term tube feeding or permanent gastrostomy or tracheotomy were not necessary during long-term follow-up.

Pre- and postoperative clinical data are displayed in Table 3.

### 3.3. Illustrative Case Reports

#### 3.3.1. Case 1

**Figure 3:**
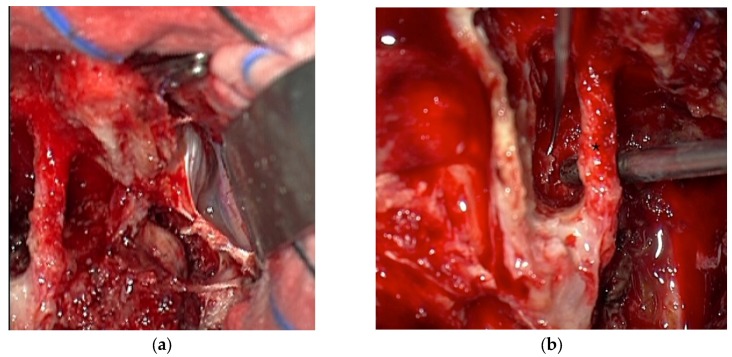
(**a**) Intraoperative image of the tumor invading the intra- and extradural jugular foramen. The sigmoid sinus is ligated; (**b**) Image of the facial nerve * within the fallopian bridge.

A 22-year-old patient presented with hearing impairment and intermittent head and neck pain. Otoscopy revealed a right vascular appearing middle ear mass. Audiometry revealed an isolated conductive deficit. Examination demonstrated tongue atrophy and deviation to one side caused by partial hypoglossal nerve palsy. No impairment of the other cranial nerves was detected. CT found a large destructive inhomogeneous right temporal bone mass extending from the upper neck and posterior fossa to the petrous apex. MRI revealed gross transdural infiltration and medullary compression. Inferiorly, the tumor extended down into the neck, invading the retro- and parapharyngeal spaces and displacing the ICA anteriorly (Figure 1). The tumor was classified as Fisch C3 D2. Genetic testing revealed SDH mutation (SDHB). Additionally, he had high levels of plasma metanephrine and normetanephrine. Preoperative superselective embolization was arranged (Figure 2). The external carotid artery was entered, and the ascending pharyngeal artery was canalized, revealing a tortuous pedicle supplying the tumor, which was embolized with ONYX. Two smaller distal pedicles of the right occipital artery and two pedicles originating from the neuromeningeal division of the vertebral artery were embolized, achieving a 70–80% reduction in tumor blush. Afterwards, the patient underwent gross total resection using a combined retro-infralabyrinthian and a lateral suboccipital approach, without facial nerve transposition (Figure 3a,b). There was a considerable intradural component encasing CNs IX to XII and compressing the brainstem. The tumor invaded the medial wall of the jugular bulb and infiltrated the lower cranial nerves, which could not be preserved. Histology revealed a malignant paraganglioma with multiple infiltrated lymph nodes. In the initial postoperative period, the patient had good vigilance but was diagnosed with a vagal lesion. On postoperative day 3, he developed severe aspiration pneumonia and required tracheostomy and ventilation for 8 days. After rehabilitation of swallowing techniques and maneuvers, he compensated the lower cranial nerve deficits well and was sent for further radiation and chemotherapy.

#### 3.3.2. Case 2

**Figure 4:**
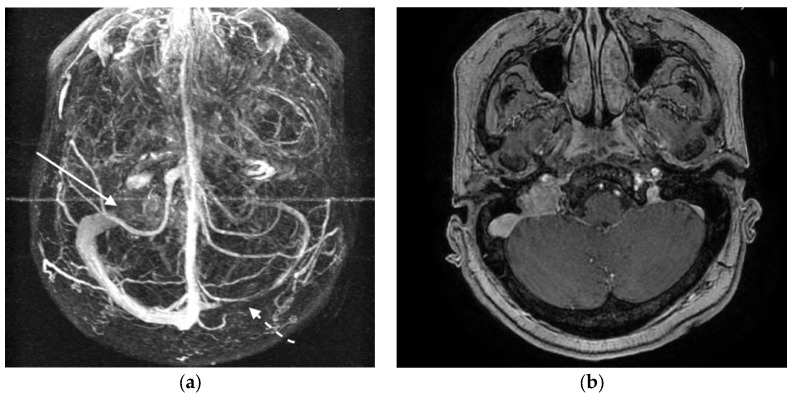
(**a**,**b**) Contrast-enhanced MRI and magnetic resonance venography demonstrating hypoplasia of the right transverse sinus (dotted arrow) and tumor invasion of the left jugular bulb (continuous arrow).

**Figure 5:**
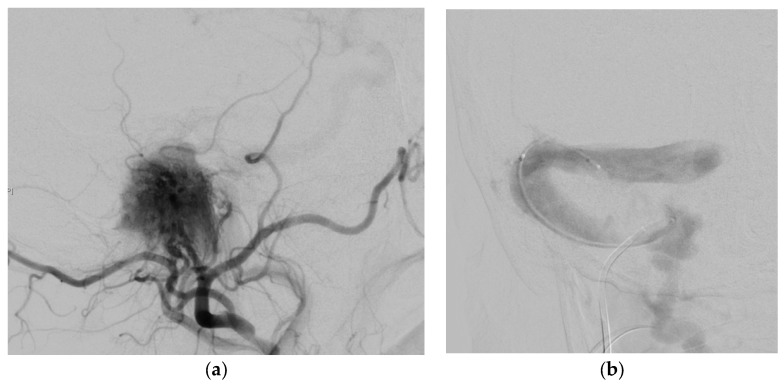
(**a**) DSA demonstrate feeding vessels from the external carotid artery; (**b**) DSA reveal reduced flow through both transverse and sigmoid sinus into the internal jugular vein.

**Figure 6:**
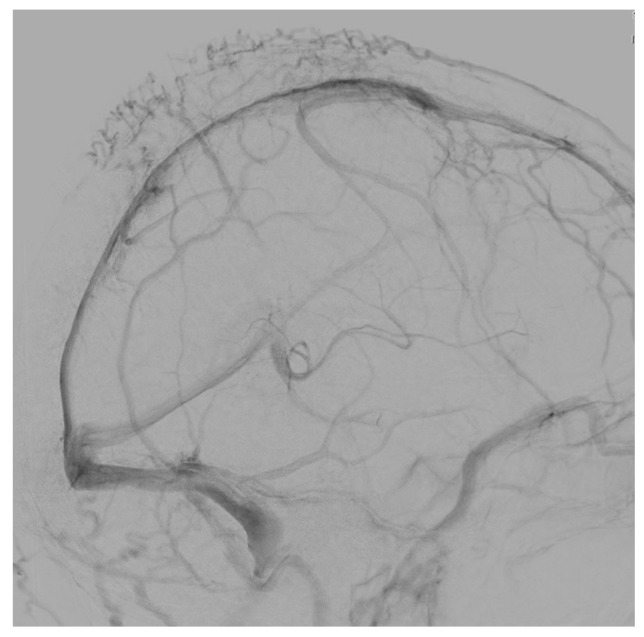
DSA reveal no flow into the internal jugular vein and many collaterals through the sagittal sinus.

A 56-year-old woman presented with a blurring of vision together with visual obscurations. Ophthalmoscopy revealed papilledema with venous engorgement, blurring of optic margins, and elevation of the optic disc. No other neurological deficits were detected. MRI demonstrated a left-sided JTP, together with suspected hypoplasia of the right transverse sinus (Figure 4a,b). DSA revealed congenitally hypoplastic right transverse and sigmoid sinuses extending into a small right internal jugular vein (Figure 4 and Figure 5). Severe focal stenosis of the distal dominant left sigmoid sinus by the tumor was observed. Direct pressure measurement in the dural venous sinuses revealed abnormally elevated pressures proximal to the stenosis and a remarkable pressure gradient across the stenosis (20 mmHg). Stent-assisted angioplasty was attempted, resulting in sinus rupture and severe subarachnoid hemorrhage. A ventriculostomy was performed to decrease intracranial pressure. She suffered from ventilation associated pneumonia and liver failure. After 10 weeks treatment in the intensive care unit, she recovered, and the external ventricular drain could be removed. Three months later, she was referred for radiation therapy. DSA demonstrated complete occlusion of the left sigmoid sinus with sufficient collateral circulation (Figure 6). One year after initial diagnosis, the visual field deficits remained stable without any deterioration.

## 4. Discussion

The clinical symptoms of patients with JTP relate to the location and extension of the tumor. Consecutively, they cause a wide variety of clinical symptoms [2,5,6,7]. Based on our experience, we propose that the treatment strategy and the surgical approach should be tailored individually to the tumor extension, the patient’s clinical symptoms and genetic testing [1,6,7,8,9,10,11].

### 4.1. Hearing Impairment and Facial Nerve Affection

The most common symptoms of JTP are progressive unilateral hearing loss caused either by impairment of vibration of the ossicles or invasion of the cochlea. Pulsatile tinnitus secondary to the tumor’s vascularity is another principal symptom of JTP.

Facial nerve palsy is a less frequent initial symptom caused by tumor infiltration in the Fallopian canal [1,12]. According to our series, if the facial nerve is infiltrated by the tumor, it is associated with postoperative facial nerve palsy. However, if the facial nerve is not infiltrated by the tumor, the risk of facial nerve dysfunction after resection of JTP cannot be eliminated. A significant incidence of transient facial nerve dysfunction depends on the surgical approach. Many authors prefer facial nerve transposition to obtain a better exposure of the tumor and the internal carotid artery (ICA) [13]. However, any degree of transposition might be associated with facial nerve palsy close to 20% [13]. Therefore, the use of facial nerve transposition is controversial. Some investigators proposed full skeletonization of the facial nerve without anterior transposition [3]. According to our experience, if the facial nerve is not infiltrated by the tumor, it is not necessary to remove it from its bony canal. We routinely performed a retro- and infralabyrinthian approach and the Fallopian-bridging technique that allows two surgical routes anterior and posterior of the facial nerve [1,13,14,15]. In case of nerve sacrifice due to tumor infiltration, and in case of complete post-operative paralysis with preserved anatomical integrity after 6 months, a combined upper eyelid loading and lower lid canthopexy provide symptomatic relief from corneal exposure with a reasonable cosmetic appearance. In case of no recovery at all, some authors perform a hypoglossus-facial nerve anastomosis [13,16]. Since surgery of large JTP is also associated with lower cranial nerve impairments and associated symptoms, these treatment options are only reserved for selected cases.

### 4.2. Lower Cranial Nerves Affection

In advanced cases, JTP grows through the medial wall of the jugular bulb into the lower cranial nerves. Distortion or invasion of the lower cranial nerves might result in dysfunctional swallowing, disturbed vocal cord function, and paralysis of the tongue. Additionally, airway protective mechanisms might be hampered by a weak cough or an insensate larynx [2,17]. Interestingly, normal cranial nerve function before surgery does not exclude tumor infiltration [18]. In these cases, the lower cranial nerves are placed at great risk during removal of large JTP. Since no recovery of LCN deficits can be expected, on long-term follow-up, these impairments result in severe patient disability, which must be carefully considered in the decision-making process. The risk is dependent on the tumor size and intradural tumor extension. Therefore, for patients with large JTP in whom the lower cranial nerve functions are intact, a wait and scan strategy or a primary radiation therapy are reasonable treatment options. If the cranial nerves seem to be invaded by the tumor, the surgeon must decide intraoperatively whether to remove the entire tumor and risk cranial nerve dysfunction or to leave a portion of the tumor on the nerves, to preserve their function. This decision must be based on the natural history of the tumor, the age of the patient, the physical condition of the patient, and the results of a detailed preoperative discussion with the patient. Younger patients and those with long-term preoperative lower cranial nerve impairment generally compensate better postoperatively, whereas patients with abrupt iatrogenic changes and older patients poorly tolerated this condition. Acute postoperative lower cranial nerve deficits result in dysphagia and aspiration. In some cases, it is necessary to provide nutrition through a nasogastric feeding tube to prevent the risk of aspiration. Temporary tracheostomy and percutaneous endoscopic gastrostomy must be performed in cases with severe aspiration pneumonia. In the next step, re-education and rehabilitation of swallowing techniques are crucial for the initial postoperative management.

### 4.3. Brainstem Compression

Tumor growth in the posterior fossa might lead to cerebellar or brainstem compression and, in some cases, to occluding hydrocephalus. Symptoms of this usually range from gait disturbance, ataxia, and hemiparesis to reduced consciousness [19]. Treatment options for large JTP with brainstem compression include total or subtotal surgical resection, radiotherapy, or a combination of the latter. The most significant effect of radiotherapy is not direct destruction of tumor cells. Furthermore, radiotherapy is related to radiation-induced fibrosis with obliteration of the vascular supply. While radiosurgery alone is appropriate for smaller tumors, in larger tumors, the potential risks of wide-field radiation are major concerns. Primary radiation of JTP with considerable brainstem compression might cause considerable swelling and a worsening of the clinical symptoms. In such cases, radiotherapy should be avoided or limited to postoperative management of the residual or progressive disease. All patients that demonstrate progressive brainstem compression should undergo surgical debulking. Similar to the results of other series symptoms associated with brainstem compression and hydrocephalus resolved after surgery [19,20].

### 4.4. Cerebral Sinus Affection

JTP regularly infiltrate the jugular bulb and obstruct the ipsilateral jugular venous outflow. In most cases, cerebral venous outflow is compensated. Hypoplasia of the transverse or sigmoid sinus contralateral to the tumor side might result in rare cases of venous congestion and, consecutively, to intracranial hypertension. Symptoms related to this condition include a chronic headache and papilledema (illustrative case 1). Less than ten such cases have been published to date [21,22]. Surgical treatment of JTP includes the closure of the sigmoid sinus and the ligation of the jugular vein. Preoperative evaluation of venous drainage of the brain is essential. When collateral venous drainage cannot be preserved or when the patient has no sufficient collateral venous drainage, a more conservative treatment strategy with preservation of the sigmoid sinus is recommended, such as radiotherapy or a wait and scan. However, if intracranial hypertension and papilledema are already present, the treatment options are limited. Stent-assisted angioplasty of the hypoplastic sinus can be attempted to reduce intracranial venous pressure [23,24]. A second step radiotherapy can also be performed.

## 5. Conclusions

In conclusion, JTP are challenging lesions. Precise clinical assessment, preoperative embolization and interdisciplinary microsurgical resection in specialized centers are the preferred treatment for selected patients. There may be also a definite role for radiotherapy in patients with preserved cranial nerve function, recurrent tumors, and patients with serious preexisting medical conditions. However, the definite treatment strategy must be based on the clinical symptoms and the results of a detailed preoperative discussion with the patient.

## Figures and Tables

**Table 1:** Adapted Fisch classification of jugulotympanic paraganglioma

Classification	Tumor Extension
Class A	Tumor limited to the middle ear cleft
Class B	Tumor limited to the tympanomastoid area with no infralabyrinthine compartment involvement
Class C	Tumor involving the infralabyrinthine compartment of the temporal bone and extending into the petrous apex
Subclass C1	Involvement of the vertical portion of the carotid canal
Subclass C2	Invasion of the vertical portion of the carotid canal
Subclass C3	invasion of the horizontal portion of the carotid canal
Subclass C4	invasion of the foramen lacerum and the cavernous sinus
Class D	Tumor with an intracranial extension
Subclass D1	Less than 2 cm in diameter
Subclass D2	Greater than 2 cm in diameter

**Table 2:** Clinical characteristics of the patients.

Clinical Characteristics	All	Observation	Radiotherapy (Alone)	Embolization	Embolization and Surgery
No. of patients	22	1	2	2	17
Age	51.3	36	78.0	74.5	49.2
Fisch type					
C2	8	1	2	2	3
C3	12	-	-	-	13
C4	1	-	-	-	1
D 1&2 (%)	88.4	0	100	100	87.7
Multiple PGL	1	-	-	-	2
Malignant PGL	1	-	-	-	1
Follow-up time (months)	81.7	24	90	80	81.3
Karnofsky at latest Follow-up	97.1	100	100	80	96.7

**Table 3:** Pre- and postoperative clinical data of 22 patients with JTP.

Symptoms	Before Treatment	After Treatment			
		Improved	Same	Worsened	New
Headache	6	3	3	-	-
Vertigo	3	1	2	-	-
Palpable cervical mass	5	5	-	-	
CN VII					
HB Grade 1	1	-	-	-	-
HB Grade 2	2	1	-	-	-
HB Grade 3	-	-	-	-	2
HB Grade 4–6	3	-	3	-	
CN VIII					
Otalgia	4	4	-	-	-
Pulsatile Tinnitus	6	5	1	-	-
Hearing deficit	9 *	3	3	-	-
Hearing loss	3	-	3	-	3 *
CN IX-X					
Hoarseness	4	-	3	1	3
Dysphagia	4	-	3	1	3
CN IX-Palsy	3	-	3	1	3
CN X-Palsy	3	-	3	1	3
CN XI-Palsy	1	-	1	-	1
CN XII-Palsy	3	-	3	-	1
Papilledema	1	1	-	-	-
Ataxia	2	2	-	-	-
Pathologic catecholeamine exprimation	1	1	-	-	-

* Three patients with preoperative hearing deficit had complete hearing loss after surgery.
